# Influence of Infill Pattern on Ballistic Resistance Capabilities of 3D-Printed Polymeric Structures

**DOI:** 10.3390/polym17131854

**Published:** 2025-07-02

**Authors:** Muhamed Bisić, Adi Pandžić, Merim Jusufbegović, Mujo Ćerimović, Predrag Elek

**Affiliations:** 1 Faculty of Mechanical Engineering, University of Belgrade, 11120 Belgrade, Serbia; pelek@mas.bg.ac.rs; 2 Faculty of Mechanical Engineering, University of Sarajevo, 71000 Sarajevo, Bosnia and Herzegovina; pandzic@mef.unsa.ba (A.P.);; 3 Faculty of Health Studies, University of Sarajevo, 71000 Sarajevo, Bosnia and Herzegovina

**Keywords:** polymer, 3D printing, defense, infill pattern, ballistic impact, penetration, experimental investigation, numerical model

## Abstract

Recent technological advances have expanded the use of 3D-printed polymer components across industries, including a growing interest in military applications. The effective defensive use of such materials depends on a thorough understanding of polymer properties, printing techniques, structural design, and influencing parameters. This paper analyzes the ballistic resistance of 3D-printed polymer structures against 9 × 19 mm projectiles. Cuboid targets with different infill patterns—cubic, grid, honeycomb, and gyroid—were fabricated and tested experimentally using live ammunition. Post-impact, CT scans were used to non-destructively measure projectile penetration depths. The honeycomb infill demonstrated superior bullet-stopping performance. Additionally, mechanical properties were experimentally determined and applied in FEM simulations, confirming the ability of commercial software to predict projectile–target interaction in complex geometries. A simplified analytical model also produced satisfactory agreement with experimental observations. The results contribute to a better understanding of impact behavior in 3D-printed polymer structures, supporting their potential application in defense systems.

## 1. Introduction

Historical evidence fully confirms the fact that since the invention of weapons, there has been a consistent tendency to develop effective protective armor. As analyzed in [1], ballistic penetration has been reported to be a critical imperil to military armor, typically to vehicles, aircraft, naval vessels, and human armor. In history, thick metal plates were widely utilized to hinder military armor from ballistic penetration. However, thick steel armor impacts mobility—armored vehicles or soldiers carrying heavy armor are slower, have reduced reaction time, waste more effort for standard movements, and can carry less payload. The urge to address these issues, which represent only a portion of a wide range of limitations, combined with the continuous advancement of armor-piercing projectiles of all calibers, has led to the strengthening of research efforts in the field of armor development. Over the centuries, the development and use of traditional manufacturing methods have been fundamental to producing armor and military tools. However, techniques have evolved to meet changing needs [2]. Understanding the historical background of various manufacturing methods in armor and military applications is crucial for seeing the bigger picture and provides a better understanding of the importance of modern methods. A review paper [3] briefly describes the technical evolution through the ages, starting with the ancient civilizations that relied on traditional blacksmithing techniques. Afterwards, in the Middle Ages, famous knight head-to-toe metal covers were crafted by shaping and tempering methods. The next big step in evolution happened during the Industrial Revolution, where assembly lines were first implemented to efficiently manufacture items such as helmets, body armor, rifles, and cannons. It is emphasized that in the 20th century, rapid advances in manufacturing technology revolutionized the production of armor and military equipment by pushing the limits with various techniques, such as stamping, forging, and welding. In addition, the capabilities of traditional manufacturing methods were improved with the introduction of new materials, such as hardened steel alloys and composite materials. By analyzing contemporary and previously mentioned methods and manufacturing techniques, it is clear that most of them, despite their historical significance, require extensive human effort and complex labor while still leaving limitations in terms of design complexity and adaptability. The constant need for better solutions, a reduction in limitations, and the pushing of boundaries has led to the increased use of additive manufacturing in the defense industry. This approach offers seemingly limitless potential for the production of advanced military equipment, providing ample room for full customization, cost reduction, less manual labor, a more environmentally friendly method compared to most others, greater operational efficiency, and more. Rooted in the fundamentals of traditional manufacturing, additive manufacturing emerges as the true answer to increasingly complex demands in the defense industry, completely transforming the landscape of military equipment production—particularly with an emphasis on protective gear, equipping armed forces with cutting-edge solutions for the future [3,4,5,6,7,8,9,10].

### 1.1. Additive Manufacturing in the Military Industry

Additive manufacturing (AM), commonly known as 3D printing, is a fabrication technology that has expanded very fast in many areas due to its many advantages, such as the adaptability, availability, and cost-effectiveness of the process when compared to the implementation of complex traditional fabrication methods. Technology that was initially imagined as only a prototyping technology soon positioned itself on a special, important level, becoming one of the most popular and advanced fabrication methods in the world [3,11,12]. AM has many different technologies based on different fabrication principles. ASTM classifies AM into seven technologies: material extrusion, material jetting, binder jetting, vat photo-polymerization, powder bed fusion, direct energy deposition, and sheet lamination [13,14,15,16,17,18,19,20]. All of these technologies are continuously improving with the capability of printing end-user products from diverse materials, including metallic and nonmetallic products [21]. Because of the numerous advantages, AM is gaining massive popularity in many industries around the globe, but one that probably stands out the most is the military industry. The military industry is very compatible with AM since it requires speed, lighter weights, and lower costs: all of the conditions that AM can work with [22]. One of the most popular AM technologies today is Fused Deposition Modeling (FDM), also known as Fused Filament Fabrication (FFF) or material extrusion technology. This method usually involves the extrusion of thermoplastic materials through a heated nozzle to create each material layer of the product [23,24,25]. The properties of FFF parts depend on process parameter selection, filament materials, and filament properties, among other factors. However, the part properties of an FFF part can only be improved to a certain extent by determining an optimum combination of process parameters through various analyses. Due to the complex geometries, different materials, and other important variations, an optimum combination of parameters is different from part to part. That is why, along with the advancements in the process parameter analysis, it is crucial to continuously search for new filament materials that can be used to produce high-quality parts to expand application areas. Researchers and industrial experts have been researching to develop new composite materials for filaments by blending different reinforcements such as particles, nanoparticles, and fibers with thermoplastics. Special focus is on composite materials, since they perform with unique properties and low costs [26,27,28,29,30,31,32,33].

### 1.2. Literature Review

By implementing the mentioned materials, the military industry adopted AM for the production of various important parts and components. So far, 3D printing is used for manufacturing different types of equipment for the military; from simple pieces and replacement parts, functional ammunition and weapons, and armor, all the way to complex structures [22,34]. Recently published review papers [35,36] provide a detailed analysis of AM (with a focus on FFF) usage in the military industry through real-life examples. Of course, it is obvious that recent world events triggered a completely new angle of AM usage for 3D printing drones, non-standardized polymer ammunition, improvised explosive devices, etc. In addition to this, there are some advanced 3D-printed weapons systems. What started with the so-called “Liberator”, the first functional 3D-printed handgun where only one bullet could be fired, escalated quickly to fully functional FFF 3D-printed weapons based on the AR15. Consisting of just a few metal parts, those guns are capable of firing hundreds of bullets. For some time now, the US military has been using large FFF 3D printers to build barracks and temporary storage facilities. One advantage of 3D-printed construction over conventional construction is the elimination of the need for formwork, which reduces material consumption, construction time, labor demand, environmental impact of materials, and cost. There are also recorded cases of 3D-printed polymer bullet casings, sabots, explosives, etc. It is certain that by understanding and developing materials for 3D printing techniques, its usage in the military industry is constantly evolving and providing infinite possibilities [35,36,37,38,39,40].

However, the main topic of this paper is the testing of FFF 3D-printed targets in order to determine the best infill pattern for potential armor use. There are recorded cases of using 3D-printed structures with various patterns combined with stronger materials (sandwich panels) as armor, and some authors in recent publications analyzed the direct impact of the infill pattern on its ballistic protection [41,42]. The study conducted in [41] provided valuable insights into the energy absorption of polymer during simulated ballistic impact with bullets of significantly lower velocity than those from a 9 mm caliber firearm. It serves as a solid foundation for further experiments on this topic, with a greater emphasis on the penetration depth of live ammunition, as explored in the present research. The aforementioned and similar studies offer excellent analyses of the ballistic resistance of various infill patterns, primarily focusing on sandwich panels, advanced engineering materials, and standardized laboratory methods. In contrast, this work employs an experimental approach involving live ammunition and the precise determination of the projectile’s stopping point (penetration depth). Although there are numerous advantages regarding the usage of 3D-printed parts, in terms of polymeric armor, it would be very inconvenient since it would require extremely large volumes and mass. As a result, recent research on modern armor has focused on exploring different combinations of materials.

The continuous need for lighter and stronger structures for space systems, aircraft, and military equipment, the development of a novel easy-to-fabricate and cost-effective sandwich panel composite stands as the most appropriate structural material. Since sandwich panels belong to the main class of structural composites, they are designed to be lightweight, possess high stiffness and strength, and exhibit remarkable energy absorption characteristics under blast or projectile impacts [43,44,45]. Sandwich panels usually consist of front and rear panels, made from tough materials such as ceramics and carbon fiber, and a middle layer, often 3D-printed polymer. The middle layer is a critical component of the sandwich structure, responsible for absorbing kinetic energy and slowing the projectile in the event that the first layer is penetrated [43]. It is important to point out that numerous experiments conducted with metal 3D-printed armor showed that structures with different infill percentages can have incredible levels of ballistic protection [46,47,48,49,50]. Infill patterns are commonly adopted by default, guided by standard evaluations of mechanical properties rather than ballistic-specific testing. This underscores the relevance of the research presented in the subsequent analysis. Despite being efficient, sandwich panels should adopt more polymer materials and become even lighter and stronger. An interesting approach for strengthening structures, besides custom infill, is described in papers [51,52] where authors analyze fish scales and osteoderms in order to replicate that protective skin mechanism as 3D-printed armor. This approach provides a lot of the inputs needed to optimize and create perfect 3D-printed armor, even when using only polymer materials. The efficiency of 3D-printed polymer armor was first confirmed in the military industry through the adoption of parts of UAVs and other important military equipment, where crucial components have been successfully protected by 3D-printed covers [3,53]. Even important sensors for direct body implementation in military monitoring applications are being 3D-printed, providing a demonstration of how durable these methods have become [54]. The direct usage of human 3D-printed armor is still in the developing phase, and a lot of work needs to be done before putting this armor in real-life situations and combats. However, 3D printing, especially in mobile ground stations directly on the field, allows for the production of custom-designed body armor parts and protective gear, offering a better fit and enhanced protection. Soldiers can also benefit from the on-site 3D printing of medical devices and prosthetics, improving medical care in remote locations. Also, an important task is trying to reduce mass and improve the mobility of military or civilian armored vehicles, so 3D printing offers a chance for a completely new way of armoring vehicles and structures [3,35,53,54,55,56]. The process is the same as 3D printing human armor, just on different scales and geometry. More examples of the combination of 3D printing and standard fabrication methods are reviewed in [57]. Previously mentioned examples of armor development and usage are from relevant scientific papers and media publications. Since this is a hot topic and relatively new, it is safe to say that the future will bring more examples of the direct implementation of this armor and its further research and development. Examples from popular media and social platforms have not been taken into consideration for this paper, as they often contain inaccurate or misleading information.

### 1.3. Aim and Scope of the Study

Taking into consideration everything from the literature review, the main objective of this paper is to analyze which type of infill pattern is the most suitable for 3D-printed polymer armor. The null hypothesis is that the combination of the most popular FFF filament—polylactic acid (PLA)—and optimal infill pattern can stop a bullet at a reasonable distance. The idea behind this work is to present a very important step on the path to creating the ultimate 3D-printed protection, relying primarily on conventional polymer materials and parameter optimization. This approach opens up possibilities for a thorough investigation of the various phenomena of the high-velocity impact and penetration of bullets or fragments from explosions. The ultimate goal is to obtain a structure as light and strong as possible while providing resistance to the aforementioned hazards for potential application in human protection and armored vehicles. An important part of this research is the experimental study, which is explained in the following chapter. The experiment was followed using a non-destructive method of determining the depth of bullet penetration through a 3D-printed PLA target. Numerical and analytical approaches are also conducted to evaluate their predictive capabilities and potentially reduce the number of required experiments. The final section provides a comprehensive analysis of the results along with a discussion of the main findings.

## 2. Materials and Methods

### 2.1. Design and 3D Printing

**Figure 1:**
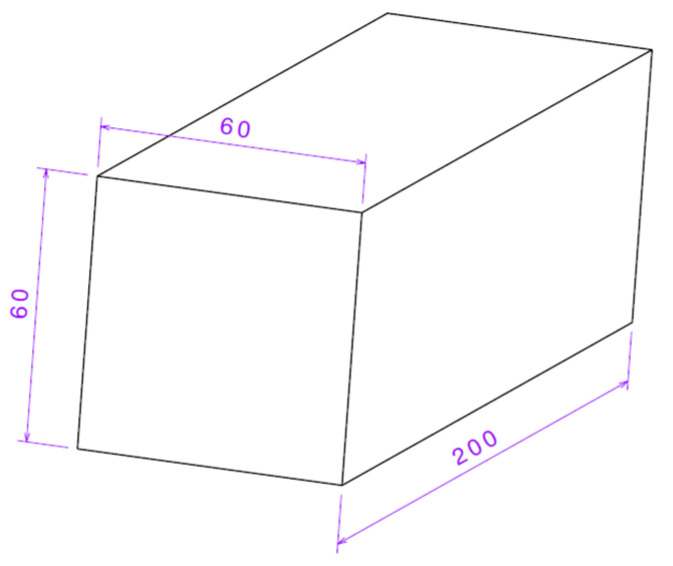
Dimensions of the constructed cuboid (dimensions in mm).

For the purpose of conducting a high-quality and replicable experiment, a cuboid with dimensions of 200 × 60 × 60 mm was designed as the target (Figure 1).

The cuboid’s depth of 200 mm was defined with the aim of retaining as many bullets as possible, and depths beyond 200 mm would not be meaningful to investigate due to the large volume. The basic hypothesis is that at least one type of infill pattern (50% density) will stop the bullet at a depth of less than 200 mm. The cuboids were designed as solid bodies, and their infill was assigned using Bambu Studio, version 1.10.1.50 (Bambu Lab, Shenzhen, China), a software intended for preparing 3D models for 3D printing, also known as a slicer.

**Figure 2:**
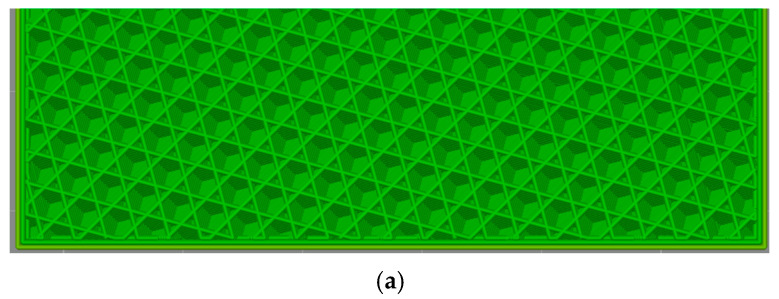

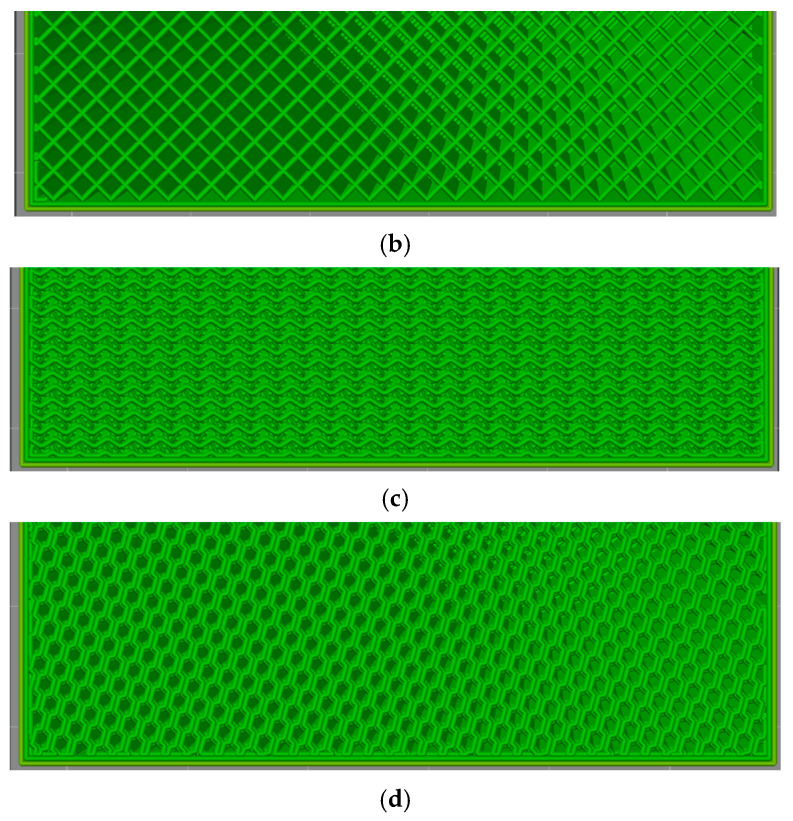
Used infill patterns: (**a**) cubic, (**b**) grid, (**c**) gyroid, and (**d**) honeycomb.

The profile used was the default 0.20 mm Standard @BBL X1C (Bambu Lab, Shenzhen, China), with the only modification being a sparse infill density of 50%, constant for all 12 cubes, and the sparse infill patterns used were cubic, grid, gyroid, and honeycomb, as seen in Figure 2.

**Figure 3:**
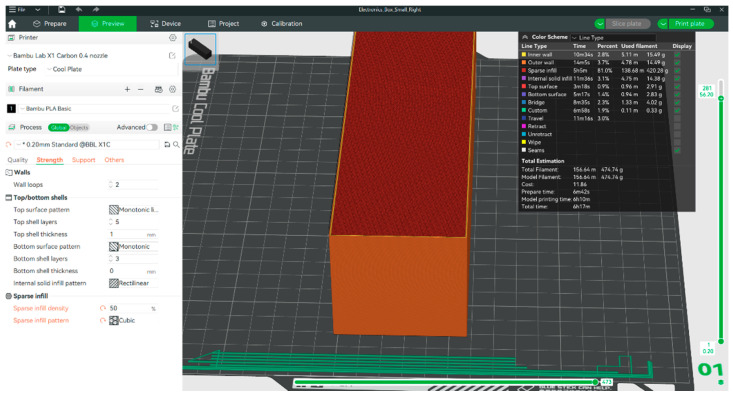
Slicing process for a cuboid with cubic infill.

An example of how a cuboid with cubic infill is sliced is shown in Figure 3.

After the G-code was generated, it was executed on a Bambu Lab X1 Carbon Combo 3D printer, using a Bambu Cool Plate coated with one layer of Bambu glue to improve material adhesion to the surface, which does not affect any properties. The main printing parameters for cuboid 3D printing are presented in Table 1 (only the infill pattern was different for each group of cuboids).

**Figure 4:**
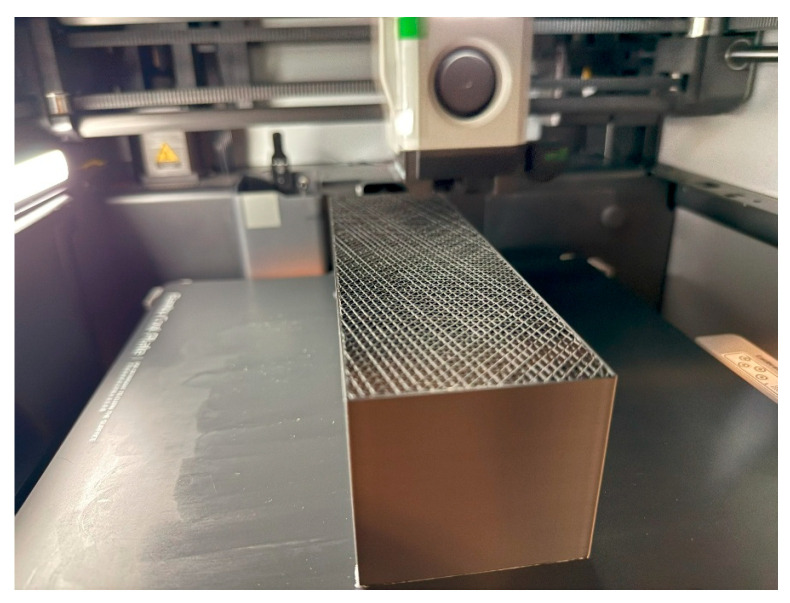
3D printing process.

The material used was Bambu PLA Basic in black for all 12 samples, due to the proven influence of color variation on material properties [58]. The orientation and position of all 12 targets were identical, as shown in Figure 4.

The average printing time and mass for the cuboid targets are listed in Table 2.

The differences in mass result from varying nozzle movements and the number of internal walls, which is not relevant in this case since all samples had the same infill density—50%.

### 2.2. Materials Tested

**Figure 5:**
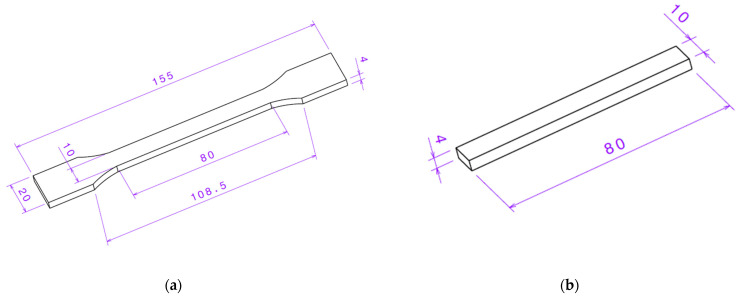
Design of specimens used for testing of (**a**) tensile properties according to ISO 527-2; and (**b**) flexural properties designed according to ISO 178 (dimensions in mm).

In this section, the tensile and flexural properties of black-colored PLA Basic 3D-printed material, manufactured by Bambu Lab, were investigated. Test specimen 3D CAD models were designed in SolidWorks 2025 (Dassault Systèmes, Vélizy-Villacoublay, France), according to the ISO 527-2 [59] standard for tensile and ISO 178 [60] for flexural mechanical properties testing, as shown in Figure 5.

All specimens were 3D-printed using FFF technology in a flat position with the Bambu Lab X1C 3D printer. The 3D printing parameters were defined in the Bambu Lab Studio slicer software V1.10.1.50, utilizing the predefined settings for the Bambu Lab black PLA Basic filament. Main printing parameters are presented in Table 3.

After 3D printing, all specimens (five for tensile and five for flexural testing) were tested according to the ISO 527 and ISO 178 standards using the Shimadzu AGS-X 10 kN universal testing machine (Shimadzu Corporation, Kyoto, Japan). The bending test was performed according to the ISO 178 standard, which specifies the use of a 3-point bending test. Based on the standards and preliminary experiments, a testing speed of 5 mm/min was applied during the experiments. The tensile and flexural properties were measured and recorded in Shimadzu Trapezium-X software version 1.5.2 (Shimadzu Corporation, Kyoto, Japan) and are presented in Table 4. The measured values were also compared with the mechanical properties of the black PLA material provided by the manufacturer in its technical data sheet (TDS) [61]. The values presented in the table represent the average of five tested specimens and show very good agreement with the manufacturer’s nominal data.

**Figure 6:**
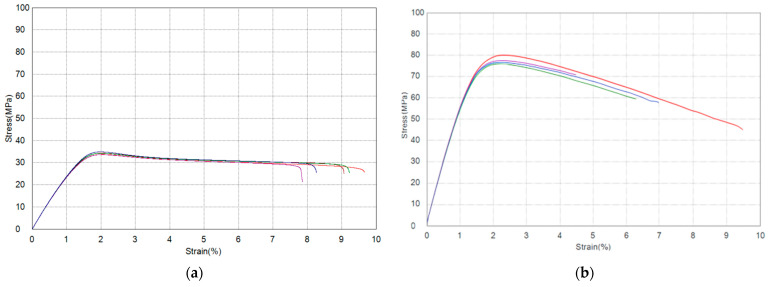
Stress–strain diagram for (**a**) tensile and (**b**) flexural properties of PLA Basic 3D-printed material.

Figure 6 presents the stress–strain curves obtained after tensile and flexural mechanical properties testing.

### 2.3. Ballistic Tests

The experiment was conducted at an outdoor shooting range at a temperature of 18 °C and cloudy weather (relevant to the valid functionality of the used chronograph). The design of the experiment ensures full repeatability, and the entire procedure is described below, accompanied by a photograph of the setup. A metal stand, anchored into the ground with an extended arm, was used to hold the cuboid target, which was secured with two layers of standard adhesive tape. In front of the stand and target, a non-branded chronograph was positioned to measure the projectile velocity. The chronograph operates by detecting the time it takes for the projectile to pass between two optical sensors spaced at a known distance. Positioned in front of the chronograph was a fixed mount holding the firearm. The distance between the gun barrel and the target was 1 m.

**Figure 7:**
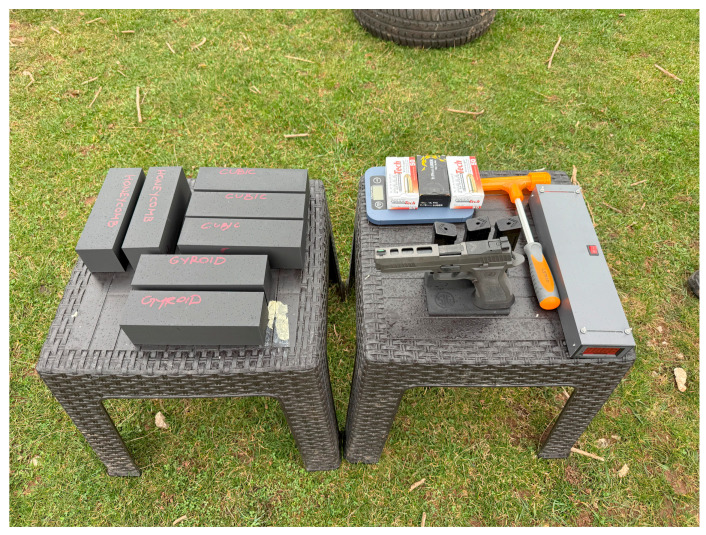
Equipment used for the experiment.

The firearm used was a Sig Sauer X-Five Legion (Sig Sauer, Eckernforde, Germany) with a 5-inch barrel. Ball 9 × 19 mm ammunition with 124-grain projectiles (mass: 8 g), manufactured by PTG (Pobjeda Technology Goražde, Goražde, Bosnia), was used. In total, there were 12 targets—3 targets per infill pattern (4 different patterns). Each of the 12 targets was hit once, each by a distinct projectile. The average measured velocity for all twelve projectiles was 355 m/s, with velocities ranging from 354 to 368 m/s, which falls within the standard range for this weapon and ammunition type. In order to check the mass of the projectile, the cartridge was carefully disassembled by using the kinetic hammer. It was confirmed that the mass matched the value specified by the manufacturer. The equipment used for the experiment is presented in Figure 7.

Each shot was fired from the fixed mount. All projectiles came from the same batch and bore the same LOT number. Only one shot was fired per target. 

**Figure 8:**
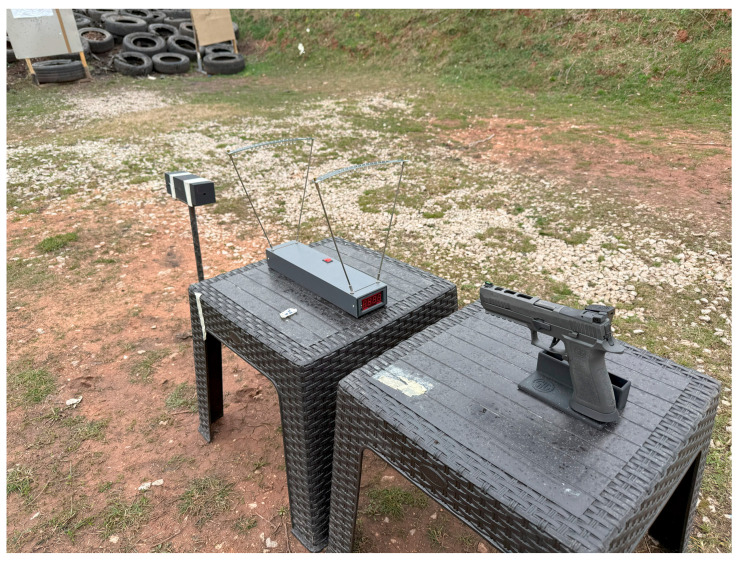
Experimental setup.

The experimental setup is shown in Figure 8.

**Figure 9:**
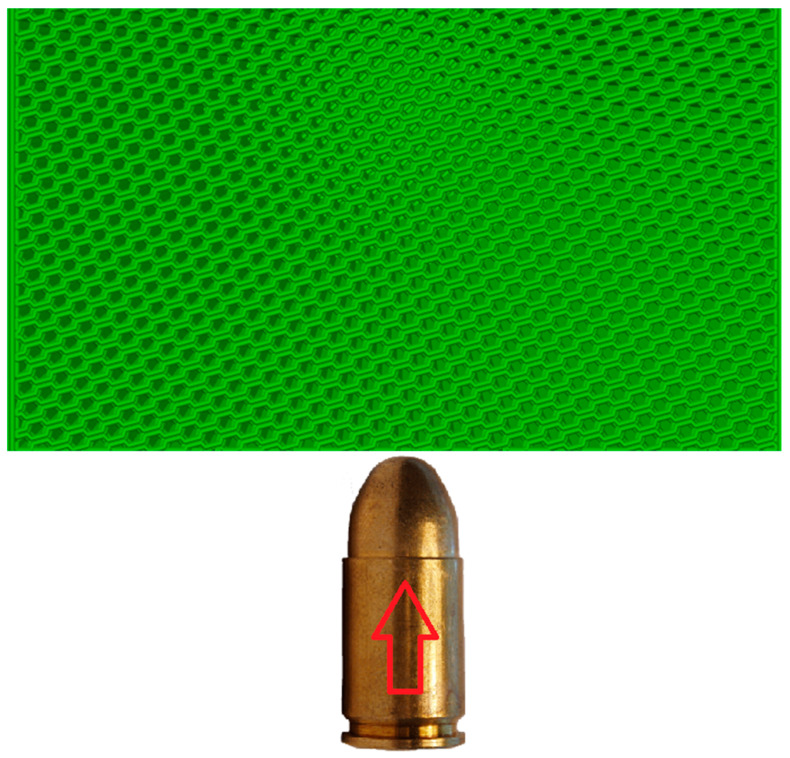
The direction of the bullet with respect to the infill pattern.

With respect to infill orientation, the projectiles were positioned to travel through the infill walls rather than striking them perpendicularly. This setup ensures that the influence of the infill pattern is a significant factor in the ballistic response, as illustrated in Figure 9.

### 2.4. Computed Tomography Analysis

The penetration depth analysis of projectiles in 3D-printed test specimens was performed using a 160-slice Canon CT Prime scanner (Canon Corporation, Minato, Tokyo, Japan). The specimens, consisting of 3D-printed cuboids with varying infill patterns (honeycomb, gyroid, grid, and cube), were scanned following ballistic testing to precisely measure the bullet penetration depth.

The CT acquisition parameters were optimized for metal artifact reduction and the clear visualization of the penetration channel. Scans were performed at a tube voltage of 120 kV with modulated mA settings, as the automatic exposure control was sufficient for the visualization of the high-contrast penetration pathways. The Single-Energy Metal Artifact Reduction (SEMAR) module was employed to minimize the beam hardening and streaking artifacts caused by the metallic projectiles, enabling a more accurate assessment of the bullet–material interface and penetration depth.

Image reconstruction was performed with a slice thickness of 0.5 mm to provide high spatial resolution for precise measurements. For optimal measurement accuracy, both coronal and sagittal multiplanar reconstructions were utilized to determine the exact location of the bullet and to measure the maximum penetration depth. This dual-plane approach ensured that measurements captured the true penetration path regardless of any deviation from the perpendicular. The penetration depths were measured from the impact surface to the deepest point of bullet penetration and recorded in centimeters. This methodology allowed for the non-destructive internal assessment of the specimens while maintaining their structural integrity for additional analyses.

### 2.5. Numerical Analysis

#### 2.5.1. General Considerations

**Figure 10:**
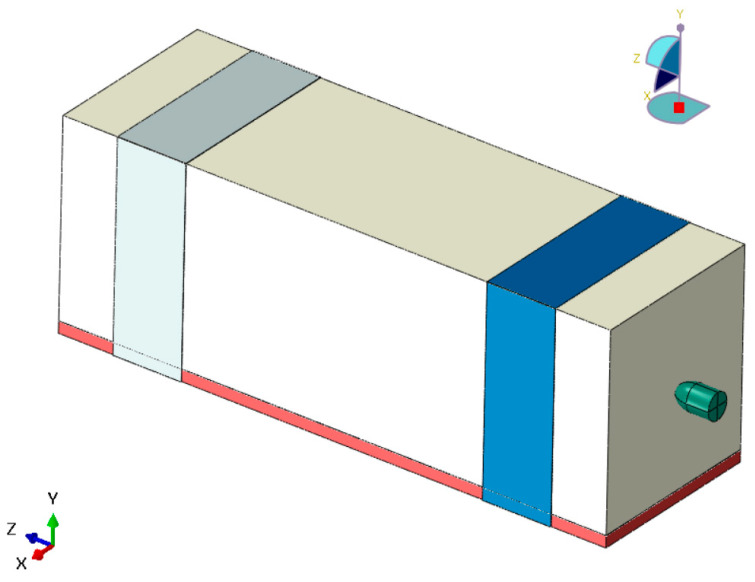
Finite element model.

A finite element model was developed in Abaqus/Explicit (6.14.4) to simulate the impact of a 9 mm bullet on a PLA polymer block, mirroring the experimental setup (Figure 10). The PLA block was positioned on a rigid steel support, and its motion was constrained by a strip of masking tape to replicate the strong adhesion observed in tests. The bullet was modeled as a rigid body (steel-jacketed lead core) with appropriate mass and inertia, while the PLA target was modeled as an orthotropic, elasto-plastic material with progressive damage and failure. Key assumptions included treating the bullet and steel support as perfectly rigid (no deformation energy absorbed by them) and neglecting gravity (due to the sub-millisecond impact duration). This section details the material models, simulation setup, and contact definitions and presents the numerical results in comparison to experimental penetration data.

#### 2.5.2. Material Models

PLA Block (3D-Printed Polymer): The PLA material was defined as an orthotropic elastic–plastic solid with damage to capture the anisotropy from the printing process and the failure under high strain rates. Elastic constants were taken from manufacturer filament data for printed PLA [61]. Table 5 summarizes the PLA properties used. The in-plane modules (E_x_, E_z_) represent the stiffness along the print raster directions, and the through-thickness modulus (E_y_) is lower due to the layer interfaces. Poisson’s ratio (ν) and shear modulus (G) were estimated based on filament data and calibrated with the tensile tests of the printed samples. Although experimental tensile tests conducted specifically on Bambu Lab PLA filament specimens exhibited plastic yielding at approximately 35 MPa with minimal post-yield hardening, a higher yield strength of 50 MPa was employed in the numerical model. This discrepancy can be rationalized by considering both the specimen size effects and the inherent strain-rate sensitivity of PLA. Under impact conditions, larger specimens typically demonstrate elevated yield strengths due to multiaxial stress states, constrained molecular deformation mechanisms, and strain-rate-induced stiffening, all phenomena well-documented in the polymer deformation literature [62,63]. A ductile damage criterion was employed: damage initiates at an equivalent plastic strain of about 5% (ε_fail_≈ 0.05), representing the onset of microcracking in PLA. The decision to use a 5% equivalent plastic strain (PEEQ) threshold for damage initiation in this numerical model is grounded in established polymer deformation and failure behavior under complex loading, rather than uniaxial tension alone [64,65]. Once damage was initiated, a linear damage evolution law was applied, with an energy dissipation (fracture energy) of the order of 2.5 kJ/m^2^, ensuring mesh-size-independent energy failure. Complete failure (damage variable D = 1) led to element deletion (erosion) and to model material separation. Section controls in Abaqus were set to allow element deletion upon failure and to enhance hourglass stiffness. Reduced-integration 8-node brick elements (C3D8R) were used for the PLA, with hourglass energy monitored to remain <5% of the internal energy (it stayed <2% in this model, indicating stable deformation modes).

Masking Tape: The constraining tape was modeled as an elastoplastic material with moderate ductility and damping characteristics to represent the tape and adhesive holding the block. The tape was given an elastic modulus of about 2.0 GPa (order of magnitude of paper/polymer laminate), a Poisson’s ratio of 0.33, and a density ~1000 kg/m^3^. The yield stress was set to 30 MPa with plastic hardening to ~40 MPa at 10% strain, allowing the tape to yield slightly without breaking (no damage/failure was assigned to the tape in the simulation, as experimentally, the tape did not rupture). Despite its minimal thickness, the masking tape was explicitly modeled using solid elements with ductile damage properties to accurately capture its role in energy dissipation and in preventing delamination, aligning closely with the observed experimental behavior [66,67].

#### 2.5.3. Model Setup

**Figure 11:**
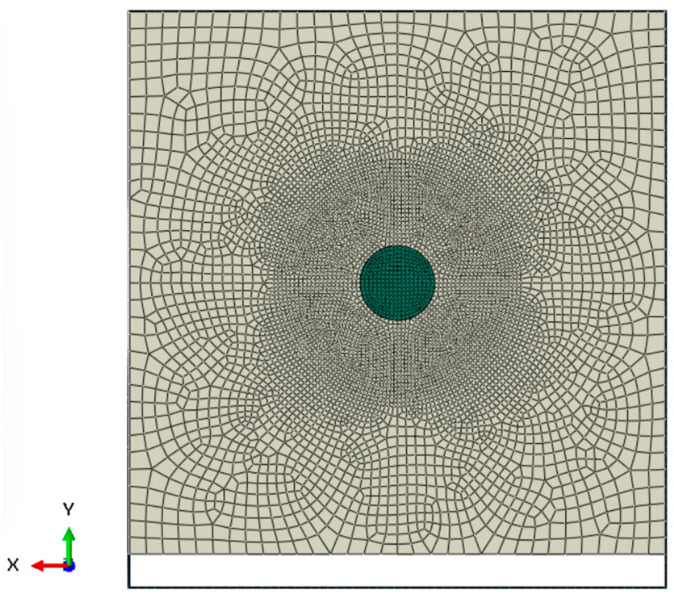
Mesh preview.

Geometry and Mesh: The PLA block was a rectangular prism with dimensions of 200 mm (length of the bullet’s travel direction) × 60 mm (width) × 60 mm (height). Due to computational constraints, the internal infill geometry was not explicitly modeled. Instead, the target was represented as an orthotropic solid, with material properties calibrated based on experimental results and manufacturer-provided filament data. Accurately modeling the real 50% infill geometry would have led to an extremely high computational cost, likely exceeding the capabilities of standard high-performance computing systems. Therefore, a trade-off between computational efficiency and geometric fidelity was necessary. Although this approach abstracts the internal structure, it provides a sufficiently accurate approximation of the macroscopic penetration behavior, ensuring the relevance of the simulation results. These dimensions ensured that the block was large enough that the bullet would be fully contained (experimentally, penetration was ~168 mm, so a 200 mm thick block provides a buffer). The block was meshed with hexahedral elements (C3D8R) using a structured mesh. A refinement was applied along the path of penetration: the region around the impact axis was meshed with ~1 mm element edges to capture the steep gradients of stress and damage, whereas outer regions used a coarser ~2 mm mesh. This graded mesh kept the total element count high but reasonable (~2,787,600 elements for the PLA block) while providing sufficient resolution in critical areas. The masking tape was represented by hexahedral elements (C3D8R) with an element size of ~0.5 mm along its length, sufficient to model its overall restraint effect. The bullet, as a rigid body, can be modeled either with a coarse mesh for contact detection or as an analytical shape; here it was meshed with elements to accurately represent its curved nose for contact. The steel support was an analytical rigid surface (a flat plate) in the simulation, so no mesh was needed for it. A Mesh preview is presented in Figure 11.

Initial and Boundary Conditions: The bullet was assigned an initial velocity of 355 m/s directed along the impact axis, replicating experimental firing conditions. Boundary conditions included a fully fixed (encastre) steel support. The PLA block was constrained by masking tape tied rigidly to the steel support and the PLA surface, effectively immobilizing the block and preventing translational and rotational motion. This ensured that the numerical model closely mimicked the experimental conditions, isolating the penetration mechanics by minimizing energy loss from unwanted movements.

Contact and Interface Modeling: The numerical model employed a hybrid contact formulation combining explicit surface-to-surface penalty contact at the initial surface contact, general contact interaction for the penetration of the primary bullet–PLA interaction, and tie constraints for secondary interfaces. The bullet surface was defined as the master surface (rigid body), and all nodes on the PLA block acted as the slave surface. Penalty-based contact controls included a defined contact stiffness scale factor (0.03) and warp–check controls (cutoff of 15 and period of 10 increments) to ensure numerical stability and minimize penetration artifacts. The friction coefficient was set to 0.35, aligning with realistic but conservative assumptions for PLA–bullet interaction [68,69,70]. The PLA block rested on a rigid steel support, modeled via frictionless penalty contact. A thin masking tape was modeled explicitly with tie constraints bonding it rigidly to both PLA and steel support surfaces, simulating adhesive bonding without cohesive failure. No contact instabilities or unrealistic penetrations occurred during simulation, confirming that the chosen hybrid contact setup effectively represented experimental conditions.

### 2.6. Analytical Considerations

This section presents an analytical approach for estimating the penetration depth of a 9 × 19 mm projectile into a 3D-printed PLA target with 50% infill density. Given the ductile nature of the target material and its significantly lower strength compared to the projectile, the penetration process occurs in the piercing or ductile hole formation regime [71,72]. The simplified model is based on the conservation of energy, assuming that the projectile’s kinetic energy is entirely dissipated through plastic deformation of the target:

(1) $$\frac{1}{2}mv^{2\;}=RP$$

where *m* and *v* are the mass and impact velocity of the projectile, *R* is the average target resistance force, and *P* is the penetration depth. The average resistance force is expressed as

(2) $$R=k_{i}k_{p}k_{d}Y_{t}A$$

Here, *k*_i_, *k*_p_, *k*_d_ are coefficients representing the influence of infill density, infill pattern, and dynamic effects, respectively. *Y*_t_ is the quasi-static compressive strength of the target material, and *A* is the cross-sectional area of the projectile, which is determined by the projectile’s diameter *d*:

(3) $$A=\frac{\pi }{4}d^{2}$$

Substituting these expressions into the energy balance Equation (1) yields the penetration depth:

(4) $$P=\frac{mv^{2}}{2k_{i}k_{p}k_{d}Y_{t}A}$$

Using known and literature-based values—projectile mass (*m* = 0.008 kg), diameter (*d* = 0.009 mm), impact velocity (*v* = 355 m/s), infill coefficient *k*_i_ = 0.5 [73], nominal pattern coefficient *k*_p_ = 1.0, a typical increase in strength due to the strain-rate effect *k*_d_ = 1.5 [74], and average quasi-static compressive strength for PLA *Y*_t_ = 68 MPa [75,76,77]—the estimated penetration depth is calculated.

## 3. Results and Discussion

This section presents all the results obtained through the previously detailed experimental, numerical, and analytical methods. To ensure clarity and systematic interpretation, the results for each method are presented and discussed individually. This approach enables a comprehensive comparison and highlights the contributions of each method to the overall analysis.

### 3.1. Experimental Results

**Figure 12:**
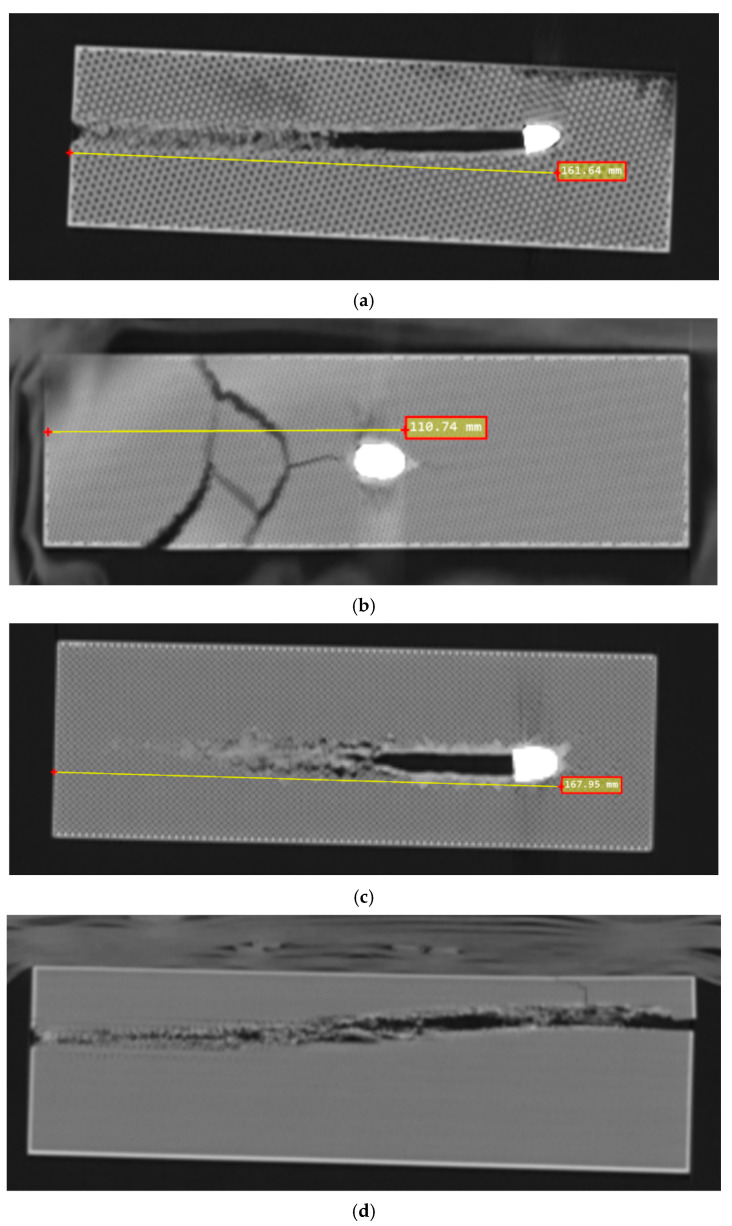
Representative CT scans for the four considered infill patterns: (**a**) cubic, (**b**) honeycomb, (**c**) grid, and (**d**) gyroid.

Given that a significant number of projectiles remained embedded in the target and their trajectories were not linear, a destructive method of measuring penetration depth would require dismantling the target and extracting the projectile. Such an approach could lead to inaccurate results due to the potential displacement of the projectile during extraction and other unintended effects. Therefore, as previously mentioned and explained, a non-destructive method using CT scanning was employed. The penetration depth of each projectile is presented in Table 6, while Figure 12 shows a representative CT scan for each type of varied infill sample.

Based on the analysis of the experimental data, the first notable and commendable observation is the consistency of the results. The fact that each infill sample produced three closely matched values, while significant differences are observed between different infill types, clearly indicates that analyzing the effect of the infill pattern is both meaningful and justified. It logically represents one of the most important steps for the further investigation of this topic.

The importance of such analysis is further supported by the fact that the gyroid infill pattern—despite showing excellent performance in mathematical modeling, impact resistance tests, and frequent use in sandwich structures—was not able to stop a bullet in a 200 mm thick target. In contrast, the cubic and grid patterns demonstrated solid stopping power. Their similar average performance is reasonable given their geometric resemblance.

As perhaps expected, the honeycomb infill exhibited outstanding capabilities, positioning itself as a promising candidate for further investigation, particularly regarding infill density variations, material selection, and other influential parameters.

### 3.2. FEM Results

**Figure 13:**
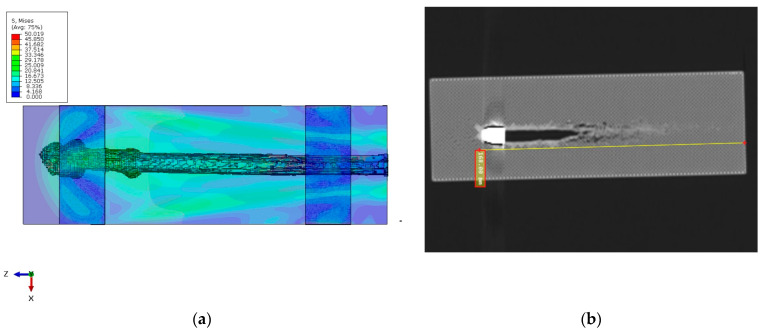
Bullet penetration: (**a**) simulation; (**b**) experimental post-mortem.

The simulation successfully captured the bullet’s penetration into the PLA block, producing a failure pattern consistent with a typical ballistic cavity. The masking tape proved crucial in maintaining the structural integrity of the block. The bullet did not perforate the block in the simulation—it came to rest inside the PLA after penetrating approximately 184 mm. This depth is in good agreement with the experimental penetration depth of ~168 mm for the grid infill pattern, differing by only about 9%. Figure 13a (simulation) and the experimental post-mortem (Figure 13b) both show that the bullet nearly traversed the block but was halted just short of an exit.

**Figure 14:**
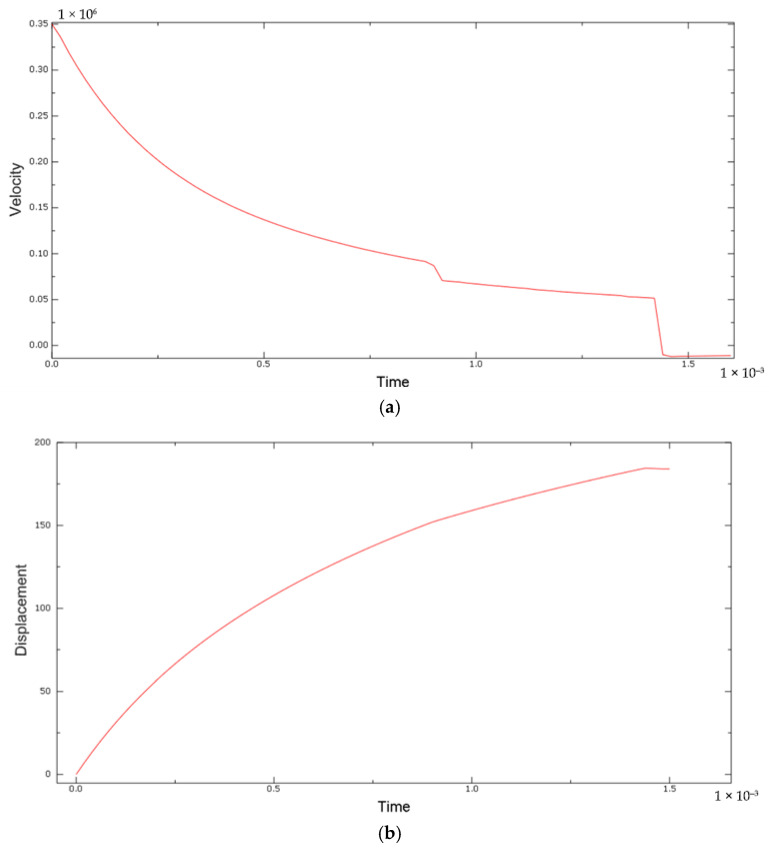
Bullet penetration numerical results: (**a**) velocity plot; (**b**) displacement plot.

The slight overestimation of penetration in the model (by ~16 mm) is attributed mainly to the idealizations in the simulation. Notably, the bullet was modeled as perfectly rigid, so it did not deform or mushroom upon impact. In reality, a 9 mm FMJ bullet may deform or yaw slightly, and energy can be absorbed in that process. Because our rigid bullet could not absorb any energy through its own deformation, more energy was available to damage the PLA, leading to a somewhat deeper penetration. Additionally, no fracture of the bullet or jacket was possible in the model, whereas experimentally, bullet fragmentation (even minimal) would also absorb energy. Velocity (a) and displacement (b) plots are presented in Figure 14.

The displacement–time response of the bullet, presented in Figure 14b, provides insights into the energy absorption behavior of the PLA block and the overall target setup. The displacement curve exhibits a smooth increase during the first 1.4 ms of the simulation, reaching a plateau at approximately 184 mm. This gradual deceleration of the bullet correlates with the absorption and dissipation of kinetic energy by the PLA material and the masking tape interface.

The curvature of the displacement profile indicates progressive resistance to penetration, characteristic of plastic deformation and local material failure. The absence of oscillations or sharp drops in the curve suggests that energy absorption occurred in a stable and distributed manner, without significant rebound or contact instabilities.

The final flattening of the curve implies that the bullet’s kinetic energy was largely dissipated by the target, consistent with the full or near-full stop observed in the numerical simulation. While kinetic energy and plastic dissipation were not explicitly extracted, the monotonic displacement trend serves as a reliable proxy for evaluating energy transfer and confirming the target’s effectiveness in halting the projectile.

Apart from the penetration depth, the qualitative damage pattern was comparable to observations. The entrance of the bullet in the simulation showed a neat 9 mm diameter puncture with the petaling of material around it, similar to the clean hole seen in the test. Internally, the simulation produced a narrow cavity of a roughly bullet-diameter for the first ~100 mm, after which it slightly expanded as the bullet slowed (the bullet’s nose pressure reduced, so less piercing and more radial cracking occurred). The terminal point of the bullet had a conical collection of heavily damaged elements around it, indicating where the bullet finally stopped. The PLA directly ahead of the bullet’s final position completely failed in the model, suggesting that the bullet was on the verge of exiting (corresponding to the slight bulge or hairline crack possibly observed on the distal side of the block in the experiment). Throughout the event, the block as a whole did not move—the tape successfully anchored it. This matches the experiment where the block remained in place on the rack. The tape in the simulation experienced some plastic strain (max ~6%) but did not fail, and it helped distribute some of the impact load into the steel support. Interestingly, the tape’s confinement caused the PLA block to behave almost like a fully clamped panel. The adhesive interface between the PLA block and the underlying steel plate was not a subject of investigation in this study, nor was it observed to influence the outcome of the penetration response. In both the physical experiment and the numerical model, sufficient masking tape coverage was applied across the top and lateral regions of the PLA block to ensure firm attachment and effective constraint. Throughout the test, no signs of debonding, tearing, or adhesive failure were observed in the tape. Visual inspection post-impact confirmed intact adhesion without cracks or delamination. This justified the modeling decision to represent the tape using *tie constraints* in the simulation, thereby enforcing perfect bonding between the PLA block, the tape, and the steel support.

The tape’s primary role was to prevent interlaminar or cohesive failure between the printed PLA layers and to fix the block in place, not to act as a variable in the energy dissipation mechanism. Since no experimental failure or interface sliding was detected, the further modeling of adhesive failure was considered unnecessary. For all tested samples, the same amount of adhesive tape and an identical mounting procedure were used to fix each 3D-printed cube to the metal fixture. Therefore, the adhesive tape can be considered a controlled variable common to all specimens and does not represent a source of variation influencing the test results. While the adhesive tape was not the primary focus of the analysis, it can be acknowledged that it contributes marginally to the overall energy dissipation during the bullet impact. The tape layer, modeled as a ductile material with plastic and damage properties, was intended primarily to constrain the PLA block and prevent interlaminar separation. However, its mechanical response inevitably results in some absorption of the projectile’s kinetic energy. Nonetheless, the presence of the tape improves the boundary constraint, reduces localized vibration at the top surface, and may slightly delay block lift-off or detachment. This enhances numerical stability and better reflects the experimental condition in which the block remained stationary. In conclusion, while the tape layer may dissipate a small portion of the impact energy, it plays a supporting role compared to the core mechanisms of contact enforcement, material plasticity, and element deletion in PLA. A more detailed breakdown of energy partitioning could be explored in future studies using dedicated sub-modeling or interface sensors.

Overall, the numerical model showed very good agreement with the experimental observations in terms of penetration depth and failure mode. The ~9.5% overshoot in penetration depth has been explained by the rigid projectile assumption. Another factor possibly contributing to the discrepancy is the exact material model parameters—the true PLA used might have slight strain-rate sensitivity (which was not included here), potentially making it a bit tougher under the high-impact strain rates. The model in this paper assumed quasi-static plastic properties; if rate hardening were included, the PLA might dissipate slightly more energy, reducing penetration. The adhesive tape’s role was clearly captured: in both the simulation and experiment, the tape prevented the block from shattering or flying off the support. Interestingly, the simulation suggests that had the tape not been present (or not been modeled), the block could have moved and perhaps resulted in less penetration (because some energy would go into motion rather than pure deformation). In the experiment, the tape essentially made the setup a fixed target, which presented replicated boundary conditions. This justifies our approach of using tie constraints for the tape.

If one were to adjust the model to perfectly match the 168 mm penetration, one could, for example, introduce a modest amount of effective bullet deformability or increase the PLA fracture energy by a small amount. However, given the complexities, a 9% difference is within an acceptable range for such high-velocity impact simulations.

The contact algorithm’s performance was good—no errant penetration or excessive distortion at the interface was seen. In summary, the numerical model is robust, and its outcomes align well with the experimental evidence of bullet penetration into a PLA block. The slight discrepancies are understood in terms of modeling assumptions (rigid bullet, ideal material properties, no projectile deformation), and the energy spike is acknowledged as a numerical artifact. These results build confidence that the model can be used to examine detailed stress-wave propagation, evaluate design changes (e.g., different tape constraints or PLA material tweaks), and provide insights into the failure processes in polymer targets under ballistic impact.

### 3.3. Analytical Calculation Results

The estimated penetration depth, based on the analytical procedure explained in detail, is calculated to be approximately 155 mm.

The analytical prediction compares reasonably well with experimental penetration depths of 162 mm and 168 mm for cubic and grid infill patterns, respectively, corresponding to deviations of 4.3% and 7.7%. These differences are primarily attributed to simplifications in the model, particularly the assumption of constant resistance force. The model does not account for the progressive crushing, thermal softening, or strain hardening of the target material.

Notably, the discrepancy between analytical predictions and experimental results is significantly larger for honeycomb and gyroid patterns—with relative errors of approximately 40% and no less than 23%, respectively—emphasizing the critical role of infill geometry.

Using the measured penetration depths, the effective *k*_i_ values inferred from the model are approximately 1.40, 0.96, 0.92, and <0.78, for the honeycomb, cubic, grid, and gyroid patterns, respectively.

While the analytical model provides acceptable results for preliminary engineering estimation, it underscores the importance of infill pattern selection and highlights areas for further refinement in predictive analytical modeling.

## 4. Conclusions

Extensive research presented in this study has confirmed the null hypothesis presented in the introduction—it is indeed possible to create a 3D-printed polymer structure capable of stopping a bullet at a reasonable distance. Through a carefully designed and executed experiment, where twelve targets were 3D-printed using the most commonly used and structurally weakest FFF polymer—PLA—with 50% infill density, nine of them successfully arrested the bullet within their volume, providing valuable data for further evaluation.

Among the four infill-type candidates often referenced in the literature for their ballistic resistance, significant variation was observed. Gyroid infill, despite its widespread use in sandwich panels, did not demonstrate any bullet-stopping capability in any of the three trials at depths of up to 200 mm. In contrast, honeycomb infill proved to be the most efficient, with an average bullet-stopping depth of 110.72 mm. Grid and cubic infills, structurally similar, yielded comparable outcomes, approximately 50% less effective than honeycomb.

Penetration depths were measured using CT scans, recognizing that only non-destructive analysis methods can provide accurate measurements in this context. All targets were fired upon under identical conditions, using a standard handgun and 9 × 19 mm live ammunition from a distance of 1 m from the muzzle.

To reduce the need for extensive experimental trials in the future and to evaluate the capability of conventional simulation tools, a series of FEM analyses was performed. These were selected due to their verified mathematical models and accessibility for potential users inspired by this study to pursue product development. A comprehensive set of material tests was conducted to validate the data from the manufacturer’s TDS. Once confirmed, these parameters were used for material modeling in the FEM software.

The results indicate that, even with the complexity of the material and manufacturing process, the FEM software—despite certain model simplifications—produced simulations with only a 9% deviation. With more detailed modeling, this deviation could be further reduced, supporting the idea that fewer experiments may be needed in the future to reach key design conclusions. Traditional analytical methods were also applied and yielded consistent results, with an average deviation of only 6%.

This alignment between experimental, numerical, and analytical methods further supports the hypothesis that it is possible to develop a stable armor solution with a predictable level of ballistic resistance. The consistency across different samples suggests that these properties are reproducible and not the result of random variation.

This research was essential, as it forms a foundation for the correct development of 3D-printed protective structures, where numerous optimal, interdependent, or independent parameters must be determined. One of the critical factors for further advancement is the selection of optimal infill geometry, as the goal is to minimize weight—i.e., reduce infill percentage—without compromising effectiveness. At 50% infill, the honeycomb pattern showed notable performance and emerged as the most promising solution. It is important to note that PLA was used in this phase, and stronger materials are expected to yield significantly better results. Identifying such optimal materials is the next step of the research. These materials must be accessible, cost-effective, and capable of withstanding ballistic impacts from fragments, projectiles, or blast waves. The confirmation of honeycomb infill as the most favorable for ballistic protection will streamline further research and accelerate progress in this field.

Since it is unrealistic to expect adequate ballistic protection using only 3D-printed polymer materials, future work will explore sandwich panels incorporating a polymer honeycomb structure as the core. Using carbon coating techniques, it is highly likely that the penetration depth of 110.72 mm measured in this study will be substantially reduced.

This represents the ultimate goal of the research: to develop a suitable and optimized armor concept for personnel, vehicles, or structural protection that is significantly lighter, more affordable, and easier to produce than conventional solutions. These are just a few of the many advantages offered by additive manufacturing technologies in the defense industry.

It is essential to continue future development steps, conduct detailed analyses, and further explore new materials and configurations for next-generation protective systems.

## Figures and Tables

**Table 1:** Printing parameters for cuboids.

FFF Printing Parameters	Value
Nozzle diameter	0.4 mm
Layer height	0.2 mm
Wall thickness	0.45 mm inner/0.42 mm outer
Infill density	50%
Printing temperature	220 °C
Printing speed	200 mm/s outer/300 mm/s inner
Build plate temperature	35 °C

**Table 2:** Printing time and target mass.

Infill Pattern	Printing Time [min]	Mass [g]
Cubic	377	474.74
Grid	371	472.26
Gyroid	1267	448.45
Honeycomb	1707	545.78

**Table 3:** FFF printing parameters used for 3D printing testing specimens.

FFF Printing Parameters	Value
Nozzle diameter	0.4 mm
Layer height	0.2 mm
Wall thickness	0.45 mm inner/0.42 mm outer
Infill pattern	Rectilinear
Infill density	100%
Printing temperature	220 °C
Printing speed	200 mm/s outer/300 mm/s inner
Build plate temperature	35 °C

**Table 4:** Comparison of measured tensile and flexural mechanical properties with the manufacturer’s values from the TDS.

Mechanical Properties	Measured Values	Values from Bambu Lab TDS
Tensile Strength [MPa]	34.1	35 ± 4
Tensile Modulus [MPa]	2600	2580 ± 220
Tensile Breaking Elongation [%]	8.8	12.2 ± 1.8
Flexural Strength [MPa]	78	76 ± 5

**Table 5:** PLA (orthotropic material) properties.

Property	Symbol	Value	Unit	Note
Density	ρ	1250	kg/m^3^	typical PLA filament density
Young’s modulus (x-direction)	E_x_	2580	MPa	transverse in-plane (print plane)
Young’s modulus (y-direction)	E_y_	2060	MPa	through thickness (layer stacking)
Young’s modulus (z-direction)	E_z_	2580	MPa	along print filament direction
Poisson’s ratio (XY plane)	υ_xy_	0.33	-	out-of-plane Poisson’s ratio
Poisson’s ratio (XZ plane)	υ_xz_	0.33	-	out-of-plane Poisson’s ratio
Poisson’s ratio (YZ plane)	υ_yz_	0.3	-	
Shear modulus (XY plane)	G_xy_	970	MPa	estimated lower due to layering
Shear modulus (XZ plane)	G_xz_	970	MPa	estimated lower due to layering
Shear modulus (YZ plane)	G_yz_	775	MPa	estimated lower due to layering
Yield stress (0.2% offset)	σ_y0_	50	MPa	onset of plastic yielding
Post-yield hardening slope	-	5	-	mild hardening to 60 MPa at 5% plastic strain
Damage initiation strain	-	0.05 (5%)	-	equivalent plastic strain at damage onset
Element deletion enabled?		Yes		elements removed at D = 1 (erosion)
Section control (hourglass)	-	Enhanced stiffness	-	improved hourglass stabilization

**Table 6:** Measured penetration depths for each target and average penetration depths.

Infill Pattern	Sample 1 [mm]	Sample 2 [mm]	Sample 3 [mm]	Average Penetration Depth [mm]
Cubic	161.64	162.41	160.50	161.51
Grid	167.95	169.12	170.55	169.20
Gyroid	>200	>200	>200	>200
Honeycomb	110.74	110.19	111.25	110.72

## Data Availability

The original contributions presented in this study are included in the article. Further inquiries can be directed to the corresponding author.

## Abbreviations

The following abbreviations are used in this manuscript:

3D	Three-dimensional
CT	Computed Tomography
FEM	Finite Element Method
AM	Additive Manufacturing
ASTM	American Society for Testing and Materials
FDM	Fused Deposition Modeling
FFF	Fused Filament Fabrication
USA	United States of America
UAV	Unmanned Aerial Vehicle
PLA	Polylactic Acid
BBL X1C	Bambu Lab X1 Carbon Combo
PTG	Pobjeda Technology Goražde
SEMAR	Single-Energy Metal Artifact Reduction
CAD	Computer-Aided Design
ISO	International Organization for Standardization
TDS	Technical Data Sheet

## References

[1] Yan J., Liu Y., Yan Z., Bai F., Shi Z., Si P., Huang F. (2022). Ballistic characteristics of 3D-printed auxetic honeycomb sandwich panel using CFRP face sheet. Int. J. Impact Eng..

[2] Marx J., Portanova M., Rabiei A. (2020). Performance of Composite Metal Foam Armors against Various Threat Sizes. J. Compos. Sci..

[3] Colorado H.A., Cardenas C.A., Gutierrez-Velazquez E.I., Escobedo J.P., Monteiro S.N. (2023). Additive manufacturing in armor and military applications: Research, materials, processing technologies, perspectives, and challenges. J. Mater. Res. Technol..

[4] Talukdar D., Dutta K. (2020). Impact of wars on the evolution of civilizations. Phys. A.

[5] Larick R. (1991). Warriors and blacksmiths: Mediating ethnicity in East African spears. J. Anthropol. Archaeol..

[6] Liao L., Pan C., Ma Y. (2010). Manufacturing techniques of armor strips excavated from Emperor Qin Shi Huang’s mausoleum, China. Trans. Nonferrous Met. Soc. China.

[7] Rodríguez Andrés A., Hempstead K. (2011). Gun control and suicide: The impact of state firearm regulations in the United States, 1995–2004. Health Policy.

[8] Allen R.C. (2017). The Industrial Revolution: A Very Short Introduction.

[9] Allen R.C. (2012). The British Industrial Revolution in Global Perspective.

[10] Harussani M.M., Sapuan S.M., Nadeem G., Rafin T., Kirubaanand W. (2022). Recent applications of carbon-based composites in defence industry: A review. Def. Technol..

[11] Colorado H.A., Mendoza D.E., Lin H.T., Gutierrez-Velasquez E. (2021). Additive manufacturing against the COVID-19 pandemic: A technological model for the adaptability and networking. J. Mater. Res. Technol..

[12] Mousa A.A., Bashir M.O. (2017). Additive manufacturing: A new industrial revolution—A review. J. Sci. Achiev..

[13] (2015). Standard Terminology for Additive Manufacturing Technologies.

[14] Braconnier D.J., Jensen R.E., Peterson A.M. (2020). Processing parameter correlations in material extrusion additive manufacturing. Addit. Manuf..

[15] Yap Y.L., Wang C., Sing S.L., Dikshit V., Yeong W.Y., Wei J. (2017). Material jetting additive manufacturing: An experimental study using designed metrological benchmarks. Precis. Eng..

[16] Ziaee M., Crane N.B. (2019). Binder jetting: A review of process, materials, and methods. Addit. Manuf..

[17] Gibson I., Rosen D., Stucker B. (2015). Additive Manufacturing Technologies: 3D Printing, Rapid Prototyping, and Direct Digital Manufacturing.

[18] Gibson I., Rosen D., Stucker B. (2015). Powder bed fusion processes. Additive Manufacturing Technologies.

[19] Ahn D.G. (2021). Directed energy deposition (DED) process: State of the art. Int. J. Precis. Eng. Manuf.-Green Technol..

[20] Gibson I., Rosen D., Stucker B. (2015). Sheet lamination processes. Additive Manufacturing Technologies.

[21] Durakovic B. (2018). Design for Additive Manufacturing: Benefits, Trends and Challenges. Period. Eng. Nat. Sci..

[22] Bisic M., Razic F., Pandzic A., Bevrnja M. (2023). Penetration testing of 3D printed projectiles made of various types of polymers. J. Mech. Sci. Technol..

[23] Pandzic A., Hodzic D., Hajro I., Tasic P. (2024). A Review of FFF 3D Printed Polymer Materials: Filaments, Physical Aspects in the Formation, Mechanical Properties and Standard Testing. Proc. 35th DAAAM Int. Symp..

[24] Cattenone A. (2018). Analysis and Simulation of Additive Manufacturing Processes.

[25] Banjanin B. (2018). Characterisation of Manufacturing Parameters of Embossing Dies Produced by 3D Printing Technique. Ph.D. Thesis.

[26] Dey A., Roan Eagle I.N., Yodo N. (2021). A Review on Filament Materials for Fused Filament Fabrication. J. Manuf. Mater. Process..

[27] Fu X., Zhang X., Huang Z. (2021). Axial crushing of Nylon and Al/Nylon hybrid tubes by FDM 3D printing. Compos. Struct..

[28] Yodo N., Dey A. (2021). Multi-Objective Optimization for FDM Process Parameters with Evolutionary Algorithms. Fused Deposition Modeling Based 3D Printing.

[29] Popescu D., Zapciu A., Amza C., Baciu F., Marinescu R. (2018). FDM process parameters influence over the mechanical properties of polymer specimens: A review. Polym. Test..

[30] Dey A., Yodo N., Khoda B. Optimizing Process Parameters under Uncertainty in Fused Deposition Modeling. Proceedings of the 2019 IIE Annual Conference.

[31] Dey A., Yodo N. (2020). Decision Analysis for Selecting FDM Process Parameters using Bayesian Network Approach. Proceedings of the 2020 IIE Annual Conference.

[32] Wickramasinghe S., Do T., Tran P. (2020). FDM-Based 3D Printing of Polymer and Associated Composite: A Review on Mechanical Properties, Defects and Treatments. Polymers.

[33] Rahim T.N.A.T., Abdullah A.M., Akil H.M. (2019). Recent Developments in Fused Deposition Modeling-Based 3D Printing of Polymers and Their Composites. Polym. Rev..

[34] Clemens M. (2022). The use of additive manufacturing in the defense sector. 3D Nativ.

[35] Marciniak M. (2023). The 3D Printing in Military Applications: FDM Technology, Materials, and Implications. Adv. Mil. Technol..

[36] Bisic M., Pandzic A., Mitrovic N., Mladenovic G., Mitrovic A. (2024). Advances in Additive Manufacturing Application in Military Industry. New Trends in Engineering Research.

[37] Pavlovich S. (2023). 3D Print Applications in Illicit Firearms Manufacture: A Review.

[38] Chandru R.A., Balasubramanian N., Oomen C., Raghunandandan B.N. (2018). Additive manufacturing of solid rocket propellant grains. J. Propuls. Power.

[39] Jagoda J., Diggs-McGee B., Kreiger M., Schuldt S. (2020). The Viability and Simplicity of 3D-Printed Construction: A Military Case Study. Infrastructures.

[40] Ma G., Zhang J., Wang L., Li Z., Sun J. (2018). Mechanical characterization of 3D printed anisotropic cementitious material by the electromechanical transducer. Smart Mater. Struct..

[41] Ma Q., Rejab M.R.M., Song Y., Zhang X., Hanon M.M., Abdullah M.H., Kumar A.P. (2024). Effect of Infill Pattern of Polylactide Acid (PLA) 3D-Printed Integral Sandwich Panels under Ballistic Impact Loading. Mater. Today Commun..

[42] Gladys A.K., Damodaran A., Ezhilan J.J., Raghavan M., Venkatesan N. (2025). Effect of Infill Patterns on Carbon Nylon 3D-Printed Composites under Ballistic Impact. Phys. Scr..

[43] Junio R.F.P., da Silveira P.H.P.M., Neuba L.d.M., Monteiro S.N., Nascimento L.F.C. (2023). Development and Applications of 3D Printing-Processed Auxetic Structures for High-Velocity Impact Protection: A Review. Eng.

[44] Callister W.D., Rethwisch D.G. (2020). Fundamentals of Materials Science and Engineering: An Integrated Approach.

[45] Li X., Zhang P., Wang Z., Wu G., Zhao L. (2014). Dynamic behavior of aluminum honeycomb sandwich panels under air blast: Experiment and numerical analysis. Compos. Struct..

[46] Rahmani R., Antonov M., Brojan M. (2020). Lightweight 3D printed Ti6Al4V-AlSi10Mg hybrid composite for impact resistance and armor piercing shielding. J. Mater. Res. Technol..

[47] Xue J., Hou Y., Chu W., Zhang Z., Dong Z., Zhang L., Wen G. (2024). 3D printed B4C-based honeycomb ceramic composite and its potential application in three-dimensional armor structure. Chem. Eng. J..

[48] (2023). Army Researchers Hack 3D Printer to Build Ceramic Body Armor. Eng. Com.

[49] Garcia-Avila M., Portanova M., Rabiei A. (2014). Ballistic performance of a composite metal foam-ceramic armor system. Procedia Mater. Sci..

[50] Medvedev A.E., Maconachie T., Leary M., Qian M., Brandt M. (2022). Perspectives on additive manufacturing for dynamic impact applications. Mater. Des..

[51] Porter M.M., Ravikumar N., Barthelat F., Martini R. (2017). 3D-printing and mechanics of bio-inspired articulated and multi-material structures. J. Mech. Behav. Biomed. Mater..

[52] Martini R., Balit Y., Barthelat F. (2017). A comparative study of bio-inspired protective scales using 3D printing and mechanical testing. Acta Biomater..

[53] Sonmez M., Pelin C.-E., Pelin G., Rusu B., Stefan A., Stelescu M.D., Ignat M., Gurau D., Georgescu M., Nituica M. (2024). Development, Testing, and Thermoforming of Thermoplastics Reinforced with Surface-Modified Aramid Fibers for Cover of Electronic Parts in Small Unmanned Aerial Vehicles Using 3D-Printed Molds. Polymers.

[54] Bird D.T., Ravindra N.M. (2021). Additive Manufacturing of Sensors for Military Monitoring Applications. Polymers.

[55] Listek V. (2023). First in-flight AM: US Marines demonstrate 3D printing aboard an MV-22 Osprey. 3DPrint.com.

[56] Gimbel E. (2020). 3D printing gains a foothold in medical and military arenas. FedTech Mag..

[57] Zou W. (2024). Recent advancements in ballistic protection—A review. J. Stud. Res..

[58] Pandzic A., Hodzic D., Milovanovic A. Influence of Material Colour on Mechanical Properties of PLA Material in FDM Technology. Proceedings of the 30th DAAAM International Symposium.

[59] (2012). Plastics—Determination of Tensile Properties—Part 2: Test Conditions for Moulding and Extrusion Plastics.

[60] (2019). Plastics—Determination of Flexural Properties.

[61] Bambu Lab (2024). Bambu PLA Basic—Technical Data Sheet.

[62] Mulliken A.D., Boyce M.C. (2006). Mechanics of the rate-dependent elastic-plastic deformation of glassy polymers from low to high strain rates. Int. J. Solids Struct..

[63] Bauwens-Crowet C., Bauwens J.C., Homès G. (1972). The temperature dependence of yield of polycarbonate in the glassy state. J. Mater. Sci..

[64] Drozdov A.D., Christiansen J.d.C. (2003). The Effect of Strain Rate on the Viscoplastic Behavior of Isotactic Polypropylene at Finite Strains. Mech. Mater..

[65] Pae K.D. (1977). The Macroscopic Yielding Behaviour of Polymers in Multiaxial Stress Fields. J. Mater. Sci..

[66] TU Delft OpenCourseWare Paper: History, Production, Properties and Products. Delft University of Technology..

[67] Kubík Ľ., Boďová I. (2016). Mechanical Properties of Crepe Paper and Chickpaper. J. Cent. Eur. Agric..

[68] Djouani F., Benlatreche Y., Djebala A. (2019). Tribological Behaviour of Polylactic Acid Against Steel Under Dry Sliding Conditions. Materials.

[69] Gohardani O., Williamson D.M., Hammond D.W. (2012). Multiple Liquid Impacts on Polymeric Matrix Composites Reinforced with Carbon Nanotubes. Wear.

[70] Shen L., Haufe J., Patel M.K. (2009). Product Overview and Market Projection of Emerging Bio-Based Plastics.

[71] Rosenberg Z., Dekel E. (2020). Terminal Ballistics.

[72] Crouch I.G. (2016). Science of Armour Materials.

[73] Mensah R.A. (2022). The effect of infill density on the fire properties of polylactic acid 3D printed parts: A short communication. Polym. Test..

[74] Priyanka G., Saideep C., Tadepalli T. (2022). Dynamic Characterization of Additively Manufactured Polylactide (PLA). Proc. Inst. Mech. Eng. Part L.

[75] Kholil A., Asyaefudin E., Pinto N., Syaripuddin S. (2022). Compression strength characteristics of ABS and PLA materials affected by layer thickness on FDM. J. Phys. Conf. Ser..

[76] Vukasovic T., Vivanco J.F., Celentano D., García-Herrera C. (2019). Characterization of the mechanical response of thermoplastic parts fabricated with 3D printing. Int. J. Adv. Manuf. Technol..

[77] Ramírez-Revilla S., Camacho-Valencia D., Gonzales-Condori E.G., Márquez G. (2023). Evaluation and comparison of the degradability and compressive and tensile properties of 3D printing polymeric materials: PLA, PETG, PC, and ASA. MRS Commun..

